# Association between neutrophil to high-density lipoprotein cholesterol ratio (NHR) and depression symptoms among the United States adults: a cross-sectional study

**DOI:** 10.1186/s12944-024-02204-y

**Published:** 2024-07-13

**Authors:** Guangwei Qing, Cheng Bao, Yuanjian Yang, Bo Wei

**Affiliations:** 1 Department of Psychiatry, Jiangxi Mental Hospital & Affiliated Mental Hospital of Nanchang University, Nanchang, 330029 Jiangxi China; 2 Third Clinical Medical College, Nanchang University, Nanchang, 330006 Jiangxi China; 3 Nanchang City Key Laboratory of Biological Psychiatry, Jiangxi Provincial Clinical Research Center on Mental Disorders, Jiangxi Mental Hospital, Nanchang, 330029 Jiangxi China

**Keywords:** Depression, Neutrophil to high-density lipoprotein cholesterol ratio (NHR), NHANES, Cross-sectional analysis

## Abstract

**Background:**

Depression acts as a noteworthy worldwide public health challenge. Identifying accessible biomarkers is crucial for early diagnosis and intervention. The relationship between depression in adult Americans and the neutrophil to high-density lipoprotein cholesterol ratio (NHR) was investigated in this research.

**Methods:**

The relationship between NHR and depressive symptoms was analyzed utilizing National Health and Nutrition Examination Survey data from 2005 to 2018 and the Patient Health Questionnaire-9. The study included 33,871 participants with complete NHR and depression data. Adjusted multivariable logistic regression models were used to account for possible confounders, and subgroup analyses were conducted to investigate effect changes.

**Results:**

Elevated NHR levels were positively correlated with a heightened risk of depression (OR = 1.03, 95% CI: 1.01–1.05, *P* < 0.0005). After the NHR was divided into tertiles, those in the top tertile had an 18% higher chance of developing depression than those in the bottom tertile (OR = 1.18; 95% CI: 1.05–1.32; *P* for trend = 0.0041). Subgroup analyses revealed variations in this association based on race and marital status. Additionally, the relationship between NHR and depression demonstrated a U-shaped pattern, with a significant breakpoint identified at an NHR of 6.97.

**Conclusion:**

These results imply that the NHR may be a potential biomarker for depression risk, with implications for early detection and personalized treatment. Further research is needed to elucidate the mechanisms underlying the NHR-depression link and establish causality.

## Background

Depression, characterized by persistent feelings of sadness, despair, and reduced self-esteem, is a prevalent psychiatric disorder leading to a lack of interest in once-pleasurable activities [1–3]. This change severely disrupts psychosocial functioning and diminishes the quality of life, adversely affecting various aspects of daily life such as work and interpersonal relationships in younger individuals [4], and significantly impairing the overall well-being and life satisfaction of older adults [5]. Globally, depression has reached epidemic levels, impacting over 300 million individuals, with a considerable portion in the Americas. By 2023, it emerged as a major contributor to global disability, alongside other mental disorders [6, 7]. This condition not only hampers individual psychosocial functioning but also burdens society immensely by straining healthcare systems, misallocating public resources, and impeding social progress and productivity [8]. Consequently, identifying accessible and predictive biological markers for depressive symptoms is vital for effective prevention, diagnosis, and treatment [9–11]. Such markers can enable healthcare professionals to tailor interventions and offer targeted support to at-risk individuals [12].

In several recent studies, the neutrophil to high-density lipoprotein cholesterol ratio (NHR) has shown potential as a biomarker [13–15]. The NHR, being a marker of both inflammation and lipid metabolism, enables a thorough analysis of these separate impacts and a more profound comprehension of their interplay, thereby shedding light on the complex mechanisms of these bodily functions [16]. Previous research has demonstrated the NHR’s remarkable predictive accuracy in various systemic conditions, including hepatocellular carcinoma, hypertension, cardiovascular risk, and acute biliary pancreatitis [13, 17–19]. While these findings highlight NHR’s sensitivity, they raise concerns about its specificity for diagnosing depression. To enhance specificity, future studies should validate NHR in combination with other biomarkers and clinical assessments for a more precise diagnostic tool. In psychiatry, NHR has shown considerable promise as a biomarker, Moreover, NHR appears to be altered differently in distinct mental disorders. For instance, the findings by Wei et al. suggest a stronger association of NHR with schizophrenia and the manic phase of bipolar disorder compared to the depressive state of bipolar disorder [20]. This distinction further highlights the need for specific validation studies on NHR’s prognostic capabilities about depression, which remains limited.

This analysis aims to explore the potential of NHR as a biomarker within the context of depression. The hypothesis that an elevated neutrophil to high-density lipoprotein cholesterol ratio (NHR) is correlated with an increased likelihood of developing depression was tested. The main goal was to use rigorous statistical approaches to account for possible confounders while analyzing cross-sectional data from the National Health and Nutrition Examination Survey (NHANES) from 2005 to 2018 to validate this hypothesis.

## Methods

### Study population

The National Health and Nutrition Examination Survey (NHANES) gives a thorough assessment of Americans’ health and nutritional status, correctly representing the country’s population, via the use of a rigorous, multistage, probabilistic sampling approach [21]. Written consent was obtained from each participant before their involvement, and this research involving human individuals was authorized by the NHANES Research Ethics Review Board. The NHANES collects a wide array of data through surveys on demographics, socioeconomic status, nutrition, and health. These are complemented by physical assessments in mobile examination centers and in-home interviews [22]. To get more information on the NHANES survey, visit the website https://www.cdc.gov/nchs/nhanes/index.htm.

**Fig. 1:**
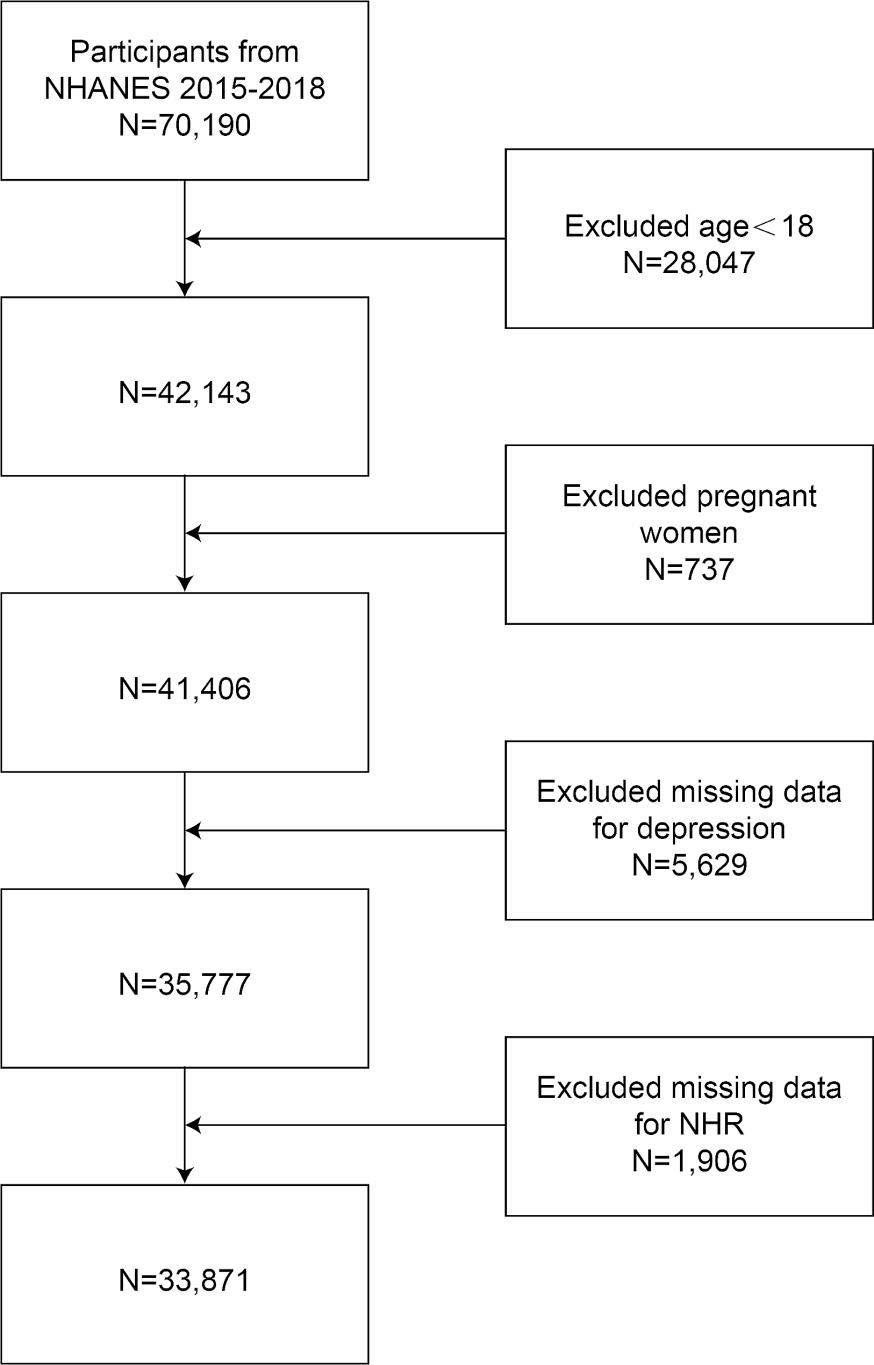
Flowchart of participant selection. NHANES, National Health and Nutrition Examination Survey

A cross-sectional approach was utilized, employing NHANES data from 2005 to 2018 and focusing on participants with complete information on NHR and depression. Of an initial pool of 70,190 individuals, those under 18 years (28,047 individuals), pregnant women (737), individuals who did not complete the Patient Health Questionnaire-9 (PHQ-9) screening for depression assessment (5,629), and those without NHR data (1,906) were excluded. The final analysis included 33,871 participants (refer to Fig. 1 for further breakdown).

### Assessment of depressive symptoms

When screening for depression, the Patient Health Questionnaire (PHQ-9) is a viable and trustworthy instrument and is widely used in primary healthcare, outpatient clinics, and research settings [23]. This nine-item measure evaluates depression symptoms that have occurred throughout the last 2 weeks [24]. A total score ranging from 0 to 27 is obtained by having participants rank each item on a scale from “0” (no incidence) to “3” (nearly daily occurrence) [25]. Scores below 10 typically indicate the absence of depression, whereas scores of 10 or higher often suggest the presence of depressive symptoms, warranting further evaluation [26]. The PHQ-9 has shown remarkable sensitivity (88%) and specificity (88%) in identifying depression [24].

### Assessment of NHR

The neutrophil count (10^3 cells/µL) divided by the high-density lipoprotein cholesterol (HDL-C) value (mmol/L), both acquired via laboratory testing, yields the neutrophil NHR [27]. For precise determination of Complete Blood Count (CBC) parameters, the Beckman Coulter DxH 800 analyzer system utilizes sophisticated counting and sizing technologies, supplemented by an automated apparatus for diluting and mixing samples, and a single-beam photometer for measuring hemoglobin. This equipment, deployed in the NHANES mobile examination centers, generates detailed CBC reports and cellular distributions for each participant, thus ensuring high precision and consistency in hematological assessments. Using enzymatic tests on Roche Modular P and Roche Cobas 6000 chemistry analyzers, HDL cholesterol levels are precisely determined, renowned for their precise biochemical measurements. These processes align with the standardized protocols specified in the NHANES CBC Profile, guaranteeing that the measurements are both accurate and replicable.

### Covariables

Data on numerous demographic and risk factors potentially affecting the relationship between depression and NHR were collected. These included age (in years), ethnic background, level of education attained, body mass index (BMI), waist circumference, poverty-to-income ratio (PIR), smoking habits, and comorbidities like diabetes, hypertension, hyperlipidemia, coronary heart disease, and stroke. White blood cell count (1000 cells/uL), individual cell counts for lymphocytes, monocytes, neutrophils, eosinophils, and basophils (1000 cells/uL), and total cholesterol (TC) concentration (mg/dL), triglyceride (TG) concentration (mmol/L), and HDL-C concentration (mmol/L) are examples of biochemical indicators. BMI was categorized as underweight (BMI < 25 kg/m²), overweight (BMI 25–30 kg/m²), or obese (BMI > 30 kg/m²). For more information on the study variables, please refer to www.cdc.gov/nchs/nhanes.

### Statistical analysis

For continuous data, means and standard deviations were computed, whereas percentages were used to represent categorical variables [28]. When comparing groups with (PHQ-9 scores ≥ 10) and without (PHQ-9 scores < 10) depression, the weighted Student’s t-test was used to find differences, and the weighted chi-square test was used for categorical variables. The correlation between NHR and depression was also explored using multivariate logistic regression analysis across three models: The three models are the unadjusted baseline (Model 1), the demographically adjusted Model 2 (adjusted for age, race, and sex), and the further adjusted Model 3 (adjusted for education, income, PIR, smoking status, diabetes, heart disease, stroke, and hypertension). This rephrasing maintains the original sentence’s technical details and meaning while adopting an objective tone for scientific reporting. To investigate the threshold effects and trends in the NHR-depression association, a two-piecewise linear regression model was used. Interaction testing and subgroup analyses were conducted across various demographic subgroups, including age, sex, educational level, race, marital status, diabetes, BMI, hypertension, hyperlipidemia, coronary heart disease, stroke, and smoking status. For statistical significance, a *P*-value of less than 0.05 was used. The ‘nhanesA’ package in the R programming language (https://github.com/cjendres1/nhanes) and the Empower Stats software (http://www.empowerstats.com) were employed for all analyses [29].

## Results

### Baseline characteristics of participants

The research cohort consisted primarily of non-Hispanic Whites, accounting for 42.60% of the total sample population. Within this subgroup, men comprised 50.17%, with an average age of 48.31 ± 18.67 years. The cohort exhibited an average depression rate of 8.97% and a mean NHR level of 3.45 ± 2.09. Notable differences were identified between individuals with and without depression, apart from age and hyperlipidemia. Those with depression were predominantly unmarried women (widowed, divorced, separated, or never married) from non-Hispanic White ethnic groups. These individuals had some college education or an associate’s degree, lower income levels, were smokers, and had hyperlipidemia and obesity. Along with greater levels of total cholesterol, triglycerides, and NHR, they also had larger numbers of white blood cells, lymphocytes, monocytes, neutrophils, eosinophils, and basophils. Nonetheless, this group did not often suffer from diabetes, hypertension, coronary heart disease, or stroke. For every relationship found, the *P*-value was less than 0.05, indicating statistical significance (Table 1).

**Table 1:** Characteristics of the study population

Characteristic	Total (*N* = 33,871)	Without depression (*N* = 30, 832)	With depression (*N* = 3, 039)	*P*-value
Age(year)	48.31 ± 18.67	48.28 ± 18.80	48.63 ± 17.27	0.236
Gender (%)				< 0.001
Male	16,993 (50.17%)	15,843 (51.38%)	1150 (37.84%)	
Female	16,878 (49.83%)	14,989 (48.62%)	1889 (62.16%)	
Race (%)				< 0.001
Mexican American	5524 (16.31%)	5034 (16.33%)	490 (16.12%)	
Other Hispanic	3236 (9.55%)	2848 (9.24%)	388 (12.77%)	
Non-Hispanic White	14,430 (42.60%)	13,160 (42.68%)	1270 (41.79%)	
Non-Hispanic Black	7112 (21.00%)	6460 (20.95%)	652 (21.45%)	
Other race	3569 (10.54%)	3330 (10.80%)	239 (7.86%)	
Marital status (%)				< 0.001
Married or living with partner	19,125 (59.06%)	17,803 (60.44%)	1322 (45.18%)	
Widowed, divorced, separated, and never married	13,258 (40.94%)	11,654 (39.56%)	1604 (54.82%)	
Education level (%)				< 0.001
Less Than 9th Grade	3260 (10.21%)	2818 (9.71%)	442 (15.23%)	
9-11th Grade	4457 (13.96%)	3862 (13.31%)	595 (20.50%)	
High school grad/GED or equivalent	7373 (23.10%)	6672 (22.99%)	701 (24.15%)	
Some college or AA degree	9481 (29.70%)	8617 (29.70%)	864 (29.76%)	
College graduate or above	7349 (23.02%)	7048 (24.29%)	301 (10.37%)	
Body mass index(kg/m^2^), (%)				< 0.001
< 25	9964 (29.71%)	9195 (30.11%)	769 (25.70%)	
25 to < 30	10,958 (32.68%)	10,178 (33.33%)	780 (26.07%)	
≥ 30	12,611 (37.61%)	11,168 (36.57%)	1443 (48.23%)	
Waist circumference(cm)	98.86 ± 16.59	98.52 ± 16.38	102.33 ± 18.29	< 0.001
Income to poverty ratio	2.50 ± 1.63	2.58 ± 1.63	1.74 ± 1.39	< 0.001
Smoking status (%)				< 0.001
Yes	14,578 (44.65%)	12,842 (43.25%)	1736 (58.67%)	
No	18,072 (55.35%)	16,849 (56.75%)	1223 (41.33%)	
Diabetes (%)				< 0.001
Yes	4231 (12.50%)	3644 (11.83%)	587 (19.35%)	
No	28,847 (85.23%)	26,493 (85.98%)	2354 (77.59%)	
Borderline	768 (2.27%)	675 (2.19%)	93 (3.07%)	
Hypertension (%)				< 0.001
Yes	11,793 (34.87%)	10,394 (33.76%)	1399 (46.14%)	
No	22,029 (65.13%)	20,396 (66.24%)	1633 (53.86%)	
Hyperlipidemia (%)				0.261
Yes	17,551 (69.54%)	16,081 (69.64%)	1470 (68.47%)	
No	7689 (30.46%)	7012 (30.36%)	677 (31.53%)	
Coronary heart disease (%)				< 0.001
Yes	1333 (4.19%)	1144 (3.95%)	189 (6.55%)	
No	30,490 (95.81%)	27,795 (96.05%)	2695 (93.45%)	
Stroke (%)				< 0.001
Yes	1213 (3.80%)	982 (3.39%)	231 (7.98%)	
No	30,691 (96.20%)	28,028 (96.61%)	2663 (92.02%)	
White blood cell count (1000 cells/uL)	7.24 ± 3.25	7.19 ± 3.29	7.72 ± 2.74	< 0.001
Lymphocyte number (1000 cells/uL)	2.19 ± 2.32	2.18 ± 2.38	2.28 ± 1.55	< 0.001
Monocyte number (1000 cells/uL)	0.56 ± 0.21	0.56 ± 0.20	0.57 ± 0.23	< 0.001
Neutrophils number (1000 cell/uL)	4.24 ± 1.76	4.20 ± 1.74	4.60 ± 1.94	< 0.001
Eosinophils number (1000 cells/uL)	0.20 ± 0.18	0.20 ± 0.18	0.21 ± 0.16	0.001
Basophils number (1000 cells/uL)	0.05 ± 0.06	0.04 ± 0.06	0.05 ± 0.06	< 0.001
HDL-C (mmol/L)	1.36 ± 0.41	1.37 ± 0.41	1.33 ± 0.41	< 0.001
Triglyceride (mmol/L)	1.40 ± 1.13	1.39 ± 1.13	1.56 ± 1.16	< 0.001
Total cholesterol (mg/dL)	191.15 ± 41.56	190.93 ± 41.35	193.43 ± 43.65	0.009
NHR	3.45 ± 2.09	3.41 ± 2.08	3.86 ± 2.15	< 0.001

### The association between NHR and depression

The multivariate regression analysis revealed a significant correlation between depression and NHR (Table 2). These findings indicate a direct relationship between a higher NHR and an increased probability of depression. Specifically, the unadjusted model showed a 10% increase in the risk of depression for every unit rise in the NHR (OR = 1.10; 95% CI: 1.08, 1.12; *P* < 0.0001). A one-unit increase in the NHR was associated with a 3% increase in the risk of depression in the complete model, and this substantial connection remained even after adjusting for all variables (OR = 1.03; 95% CI: 1.01, 1.05; *P* < 0.0005). A sensitivity analysis using adjusted models and categorizing NHR levels into tertiles showed that, in the unadjusted model, Tertile 3 (highest NHR) had an 18% greater likelihood of depression than Tertile 1 (lowest NHR) (OR = 1.18; 95% CI: 1.05, 1.32; *P* for trend = 0.0003). Within the model with partial adjustments, Tertile 2 demonstrated a 12% higher risk of depression than Tertile 1 (OR = 1.12; 95% CI: 1.02, 1.24; *P* = 0.0001). However, there were no significant differences found between Tertiles 1 and 2 in the crude and fully adjusted models (Table 2).

**Table 2:** The association between NHR and depression

	Crude model (Model 1)	Partially adjusted model (Model 2)	Fully adjusted model (Model 3)
	OR (95% CI) *p*-value	OR (95% CI) *p*-value	OR (95% CI) *p*-value
NHR	1.10 (1.08, 1.12) < 0.0001	1.12 (1.10, 1.15) < 0.0001	1.03 (1.01, 1.05) 0.0005
NHR Tertiles			
Tertile 1 (<2.43)	Reference	Reference	Reference
Tertile 2 (2.43 to <3.80)	1.05 (0.95, 1.16) 0.3174	1.12 (1.02, 1.24) 0.0215	0.93 (0.83, 1.04) 0.2250
Tertile 3 (≥ 3.80)	1.60 (1.46, 1.75) < 0.0001	1.83 (1.66, 2.01) < 0.0001	1.18 (1.05, 1.32) 0.0041
*P* for trend	1.17 (1.14, 1.20) < 0.0001	1.22 (1.18, 1.26) < 0.0001	1.07 (1.03, 1.10) 0.0003

Model 1, no covariates were adjusted. Model 2, age, gender, and race were adjusted. Model 3, age, gender, race, marital status, education level, BMI, income-to-poverty ratio, smoking status, diabetes, coronary heart disease, stroke, and hypertension were adjusted. *95% CI* 95% confidence interval, *OR* odds ratio

### Nonlinear relationship between NHR and depression

**Fig. 2:**
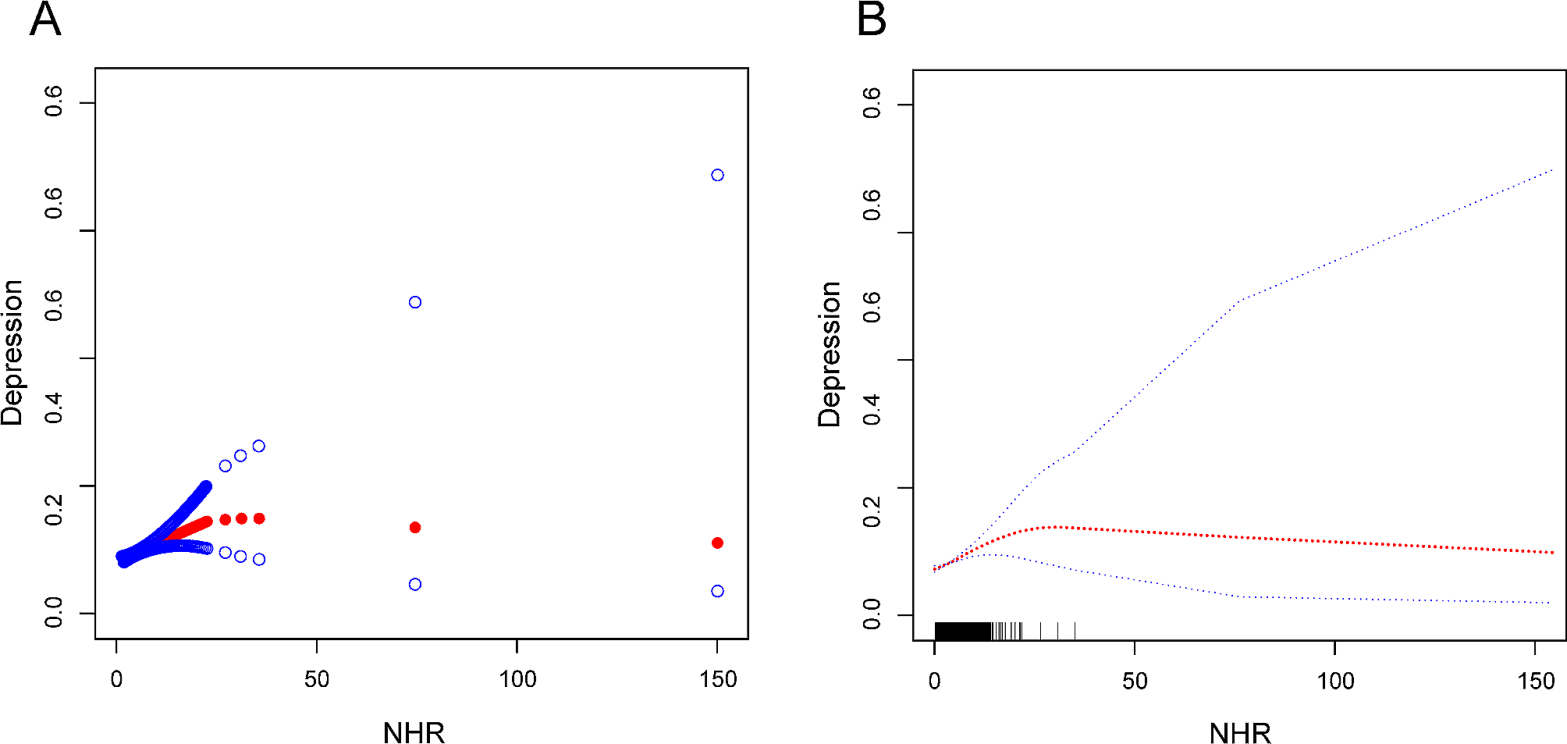
The association between NHR and depression. (**A**) The solid red line represents the smooth curve fit between variables. (**B**) Blue bands represent the 95% confidence interval from the fit

Using advanced statistical methodologies, to evaluate the complex nonlinear link between NHR levels and depression, a thorough investigation was carried out. By employing smooth curve-fitting techniques and weighted generalized additive models, a significant nonlinear association between NHR levels and the frequency of depression was identified. Further analysis using a two-segment linear regression model revealed a nonlinear curve that describes the relationship between NHR and depression (Fig. 2). A statistically significant P-value of 0.005 was obtained using a logarithmic likelihood ratio test that revealed a breakpoint of 6.97 (Table 3).

**Table 3:** The threshold effect of NHR on depression was analyzed using a two-part linear regression model

Depression	Model: saturation effect analysis
Fitting by the standard linear model	
OR (95% CI)	1.03 (1.01, 1.05)
*P*-value	0.0005
Fitting by two-piecewise linear model	
Breakpoint (K)	6.97
OR1(< K)	1.07 (1.04, 1.10) < 0.0001
OR2(> K)	1.01 (0.98, 1.04) 0.6718
Logarithmic likelihood ratio test *P*-value	0.005

Model 1, no covariates were adjusted. Model 2, age, gender, and race were adjusted. Model 3, age, gender, race, marital status, education level, BMI, income-to-poverty ratio, smoking status, diabetes, coronary heart disease, stroke, and hypertension were adjusted. *95% CI* 95% confidence interval, *OR* odds ratio

### Subgroup analysis

The subgroup analysis aimed to identify potential disparities in the association between depression and NHR levels among different demographic and clinical subpopulations to identify influencing factors or markers useful for stratification. Participants were categorized according to age groups (< 50 and ≥ 50), sex, race, BMI categories (< 25, 25–30, > 30), educational attainment, marital status, and the presence of comorbidities such as hypertension, hyperlipidemia, diabetes, coronary heart disease, history of stroke, and smoking status. Subgroup analyses and interaction tests were conducted as well. These findings indicated variations in the observed correlations, particularly with statistically significant disparities based on race and marital status (*P* < 0.05). Regarding the other demographic groupings, no significant differences were noted. These results suggest that race and marital status influence the relationship between NHR and depression, emphasizing the need to consider these variables in future studies to achieve greater precision. Additionally, the data revealed that the association between depression and NHR levels remained consistent across sex, age, BMI, education level, and various health condition groups (Table 4).

**Table 4:** Association between NHR and depression in subgroups

Subgroup		OR(95%CI)	*P* for interaction
Age(year)			0.2375
< 50	*N* = 14,478	1.05 (1.02, 1.08)	
≥ 50	*N* = 14,556	1.03 (1.01, 1.05)	
Gender			0.3728
Male	*N* = 14,536	1.03 (1.01, 1.05)	
Female	*N* = 14,498	1.04 (1.01, 1.07)	
Race			0.0443
Mexican American	*N* = 4415	1.00 (0.96, 1.04)	
Other Hispanic	*N* = 2645	1.06 (0.99, 1.14)	
Non-Hispanic White	*N* = 13,033	1.06 (1.03, 1.09)	
Non-Hispanic Black	*N* = 5921	1.02 (0.98, 1.06)	
Other race	*N* = 3020	1.08 (1.00, 1.16)	
BMI (kg/m2)			0.1318
< 25	*N* = 8225	1.03 (0.99, 1.07)	
25–30	* N* = 9603	1.02 (1.00, 1.04)	
> 30	*N* = 11,206	1.06 (1.03, 1.09)	
Education level			0.1784
Less than 9th grade	*N* = 2754	1.08 (1.01, 1.15)	
9-11th Grade	*N* = 3991	1.04 (1.00, 1.08)	
High school grad/GED or equivalent	*N* = 6711	1.05 (1.01, 1.10)	
Some college or AA degree	*N* = 8739	1.01 (0.99, 1.04)	
College graduate or above	*N* = 6826	1.06 (1.00, 1.13)	
Marital status			0.0099
Married/living with partner	*N* = 17,420	1.07 (1.04, 1.10)	
Widowed/divorced/separated/ Never married	*N* = 11,606	1.02 (1.00, 1.04)	
Hypertension			0.2763
Yes	*N* = 10,547	1.03 (1.01, 1.05)	
No	*N* = 18,446	1.05 (1.02, 1.08)	
Hyperlipidemia			0.8709
Yes	*N* = 15,846	1.03 (1.01, 1.05)	
No	*N* = 6596	1.03 (1.00, 1.07)	
Diabetes			0.0653
Yes	*N* = 3750	1.08 (1.03, 1.13)	
No	*N* = 24,579	1.02 (1.01, 1.04)	
Borderline	*N* = 690	1.10 (0.98, 1.24)	
Coronary heart disease			0.2494
Yes	*N* = 1197	1.09 (1.00, 1.18)	
No	*N* = 27,744	1.03 (1.01, 1.05)	
Stroke			0.6017
Yes	*N* = 1068	1.05 (0.98, 1.12)	
No	*N* = 27,935	1.03 (1.01, 1.05)	
Smoking status			0.1253
Yes	*N* = 13,264	1.05 (1.02, 1.07)	
No	*N* = 15,756	1.02 (1.00, 1.04)	

The results show that the subgroup analysis was adjusted for all presented covariates except theeffect modifier. *95% CI* 95% confidence interval, *OR* odds ratio

## Discussion

An analysis of the relationship between NHR and depressed symptoms was conducted by applying data from the NHANES database, a nationally representative survey of the US population from 2005 to 2018. A statistically significant correlation between raised NHR levels and a higher risk of getting depression was discovered via thorough statistical research. The data revealed a nonlinear positive association, with distinct patterns for NHR levels below and above the critical breakpoint of 6.97.

Drawing on existing data, this study represents a pioneering effort to explore the association between NHR and depression—however, the broader utility of the NHR as a peripheral trait biomarker in mental health. Limited research exists, with only one retrospective study specifically examining the NHR’s prognostic importance in schizophrenia and bipolar disorder rather than depression. Wei et al. conducted a cross-sectional study involving 13,329 patients with schizophrenia and 6,005 patients with bipolar disorder hospitalized between January 2015 and January 2021 [20]. Through the examination of sociodemographic and hematological information taken from an electronic health record, they demonstrated that the NHR can be used to assist in the identification and differential diagnosis of patients with schizophrenia and bipolar disorder in the manic phase.

Although previous research has not specifically focused on the relationship between NHR and depression, recent investigations have explored the connection between depression and other hematological markers, particularly those related to neutrophils and HDL-C. According to recent research by Meng et al., the NLR exhibited a significant and complex nonlinear association with depression, even after accounting for potential confounders [30]. Using data from the NHANES, their comprehensive cross-sectional study evaluated 34,324 individuals, revealing that 3009 of them had been diagnosed with major depression. Shin et al. carried out a cross-sectional investigation to investigate HDL-C levels in a representative group of 8,207 Koreans aged between 40 and 64 [31]. Through a rigorous investigation, they discovered a linkage between higher HDL-C concentrations and an amplified susceptibility to depressive symptoms in this specific middle-aged Korean population. Furthermore, Chen and colleagues investigated a novel inflammatory marker, the lymphocyte-to-high-density lipoprotein ratio (LHR), in a representative sample of 4,216 Americans using NHANES data [32]. Through their investigation, they uncovered a significant association between elevated LHR values and a heightened likelihood of depression development. This research aligns with these studies, indicating a direct correlation between hematologic indicators involving NHR and an increased likelihood of depression.

Several potential pathways are essential for comprehending the relationship between NHR and depression, even if the precise processes underlying this correlation are still unclear. The NHR, known for its role as an inflammatory biomarker, is believed to influence psychological states and behavior through cytokine-mediated pathways, potentially affecting the central nervous system (CNS) [33, 34]. Elevated cytokine levels due to increased neutrophil activity are thought to substantially affect the CNS [35, 36]. Upon entering the brain, these cytokines can intensify CNS inflammation and provoke a neuroinflammatory response, particularly when interacting with microglia [37, 38]. This inflammation is associated with the dysregulation of neurotransmitters, such as reduced serotonin levels and increased dopamine turnover, both of which are vital for mood regulation [39, 40]. Additionally, HDL-C interacts with endothelial cell membranes, reducing their activation and the expression of inflammatory mediators [41]. HDL-C also has antioxidant properties, neutralizes free radicals, and diminishes oxidative stress [42]. Since oxidative stress can trigger inflammatory pathways, the antioxidant function of HDL-C may indirectly reduce inflammation [43]. This decrease in inflammation can subsequently mitigate the negative effects of inflammatory cytokines on the CNS, thereby modulating mood disturbances linked to depression [44]. Moreover, HDL-C is increasingly recognized for its neuroprotective benefits [45]. It is essential for preserving the integrity of the blood-brain barrier as well as the makeup and operation of lipid rafts [46, 47]. These structures are essential for neuronal plasticity, which is crucial for operating neurotransmitter receptors and transmitting neural signals [48]. Lipid rafts also regulate various intracerebral signaling pathways, which are fundamental for emotional regulation and the maintenance of cognitive function maintenance [49].

It is noteworthy that NHR levels were reported to be higher in unipolar depression with psychotic symptoms than in those without by Wei et al. This research raises the possibility that psychotic and non-psychotic states vary biologically. In the study, depressive symptoms were significant even compared to individuals with only mild symptoms or no symptoms at all, highlighting the relevance of NHR in detecting varying degrees of depressive symptoms in the general population. Psychotic disorders or symptoms may be associated with even more disrupted inflammatory pathways [50], contributing to the observed differences between psychotic and non-psychotic states. Psychotic symptoms might exacerbate inflammatory responses, leading to higher levels of pro-inflammatory cytokines [51]. This heightened inflammatory state could further disrupt neurotransmitter systems, crucial for mood regulation, and perpetuate a cycle of neuroinflammation. The chronic stress often associated with psychotic disorders can also perpetuate inflammation [52], potentially explaining the elevated NHR levels observed in these conditions.

### Strengths and limitations

This research offers several significant advantages. Notably, a nationally representative sample that represents the demographic variety of the adult U.S. population is used, making it noteworthy that it leads the investigation of the relationship between the NHR and the risk of depression. This extensive and varied sample base enhances the external validity of the findings. Additionally, the robustness of this study is bolstered by meticulous subgroup analysis and the adjustment of critical covariates, which substantiates the reliability of conclusions. Emphasis was also placed on investigating the non-linearity of the NHR-depression link and conducting threshold analyses, providing a more nuanced understanding of the complex interplay between these variables.

Nonetheless, it is critical to recognize the limitations that this research has. Firstly, the cross-sectional design makes the results susceptible to the possibility of reverse causality, as it provides a single-instance assessment rather than a longitudinal perspective. Secondly, the reliance on self-reported questionnaires rather than in-depth psychiatric evaluations introduces the potential for recall bias and response bias. It may not fully capture clinical diagnoses of depression [53]. Thirdly, the conclusions cannot be broadly applied to other groups since the relevance of these findings is limited to the American demography. Fourthly, the study cohort consisted of participants from the general population, not individuals with clinically diagnosed psychiatric conditions. Thus, it is unclear if the observed associations between NHR and depressive symptoms apply to a clinical population with major depressive disorder. Fifthly, we lack detailed information on the duration and stage of depressive episodes at the time of NHR measurement, which is crucial for understanding the relationship between NHR and depression severity. Future studies should include clinically diagnosed patients and detailed episode information. Sixthly, this study does not elucidate the underlying molecular mechanisms that associate the observed NHR with the risk of depression, thereby limiting our understanding of the causal relationship. Seventhly, an exclusive focus on NHR as a putative biomarker for depression risks neglecting other significant biomarkers or marker combinations that could offer a more holistic appraisal of the risk profile for depression. Eighthly, despite accounting for numerous significant confounding factors, there remains a risk that unmeasured confounders, such as the age of onset for depression, the chronicity of depressive episodes, and specifics regarding the use of antidepressant medications, may influence the outcomes [54]. Ultimately, while a significant nonlinear relationship is identified, the clinical implications necessitate further exploration for practical application in clinical settings [55–57].

## Conclusion

This research identified a significant link between high NHR levels and an increased likelihood of depression, underscoring NHR’s potential as a novel biomarker for mental health conditions. This highlights its importance in identifying depression and tailoring patient-specific treatment strategies.

## Data Availability

In this study, publicly accessible datasets were examined. These data can be found here: (https://wwwn.cdc.gov/nchs/nhanes/analyticguidelines.aspx, accessed on 1 November 2022).

## Abbreviations

HDL-C: High-density lipoprotein cholesterol
NHR: Neutrophil to high-density lipoprotein cholesterol ratio
PIR: Poverty-to-income ratio
BMI: Body mass index
TC: Cholesterol

## References

[1] Huang AA, Huang SY (2023). Increased vigorous exercise and decreased sedentary activities are associated with decreased depressive symptoms in United States adults: analysis of the National Health and Nutrition Examination Survey (NHANES) 2017–2020. Health Sci Rep.

[2] Huang AA, Huang SY (2023). Quantification of the Effect of Vitamin E Intake on depressive symptoms in United States adults using restricted cubic splines. Curr Dev Nutr.

[3] Huang AA, Huang SY. Increasing Potassium Intake Up to 2300 mg is Associated with Decreased Depressive Symptoms in United States Adults: analysis of the National Health and Nutrition Examination Survey (NHANES) 2017-2020. October 17, 2022. 10.1002/hsr2.1473 PMC10405577 37554955

[4] Thapar A, Eyre O, Patel V, Brent D (2022). Depression in young people. Lancet.

[5] Sivertsen H, Bjørkløf GH, Engedal K, Selbæk G, Helvik AS (2015). Depression and Quality of Life in older persons: a review. Dement Geriatr Cogn Disord.

[6] Yin J, Li S, Li J, Gong R, Jia Z, Liu J, Jin Z, Yang J, Liu Y (2023). Association of serum oleic acid level with depression in American adults: a cross-sectional study. BMC Psychiatry.

[7] Malhi GS, Mann JJ (2018). Depression. Lancet.

[8] Stecher C, Cloonan S, Domino ME. The Economics of Treatment for Depression. Annu Rev Public Health 2023. 10.1146/annurev-publhealth-061022-040533 38100648

[9] Davis E, Lee S (2023). The relationship between omega-3 fatty acid intake and depression: a longitudinal study. Nutr Neurosci.

[10] Smith J, Doe A (2023). The impact of diet on mental health: a systematic review. J Nutr Mental Health.

[11] Brown KL, Patel SR (2023). The role of physical activity in reducing symptoms of depression: a meta-analysis. Am J Prev Med.

[12] Johnson LM, Wong RT (2022). Machine learning applications in predicting cardiovascular diseases: a review. Int J Cardiol.

[13] Shi K, Hou J, Zhang Q, Bi Y, Zeng X, Wang X (2023). Neutrophil-to-high-density-lipoprotein-cholesterol ratio and mortality among patients with hepatocellular carcinoma. Front Nutr.

[14] Wei Y, Gao H, Luo Y, Feng J, Li G, Wang T, Xu H, Yin L, Ma J, Chen J (2024). Systemic inflammation and oxidative stress markers in patients with unipolar and bipolar depression: a large-scale study. J Affect Disord.

[15] Korkmaz ŞA, Kızgın S (2023). Neutrophil/high-density lipoprotein cholesterol (HDL), monocyte/HDL and platelet/HDL ratios are increased in acute mania as markers of inflammation, even after controlling for confounding factors. Curr Med Res Opin.

[16] Jiang M, Sun J, Zou H, Li M, Su Z, Sun W, Kong X (2022). Prognostic role of neutrophil to high-density lipoprotein cholesterol ratio for all-cause and Cardiovascular Mortality in the General Population. Front Cardiovasc Med.

[17] Zhang Y, Ding Y, Jiang W (2023). Neutrophil and monocyte ratios to high-density lipoprotein cholesterol as biomarkers in non-dipping hypertension. Clin Exp Hypertens.

[18] Pan X, Zhang X, Ban J, Yue L, Ren L, Chen S (2023). Association of Neutrophil to High-Density Lipoprotein Cholesterol Ratio with Cardiac Ultrasound parameters and Cardiovascular Risk: a cross-sectional study based on healthy populations. J Inflamm Res.

[19] Yang L, He C, Wang W (2024). Association between neutrophil to high-density lipoprotein cholesterol ratio and disease severity in patients with acute biliary pancreatitis. Ann Med.

[20] Wei Y, Wang T, Li G, Feng J, Deng L, Xu H, Yin L, Ma J, Chen D, Chen J (2022). Investigation of systemic immune-inflammation index, neutrophil/high-density lipoprotein ratio, lymphocyte/high-density lipoprotein ratio, and monocyte/high-density lipoprotein ratio as indicators of inflammation in patients with schizophrenia and bipolar disorder. Front Psychiatry.

[21] Paulose-Ram R, Graber JE, Woodwell D, Ahluwalia N (2021). The National Health and Nutrition Examination Survey (NHANES), 2021–2022: Adapting Data Collection in a COVID-19 environment. Am J Public Health.

[22] Hoffman HJ, Rawal S, Li CM, Duffy VB (2016). New chemosensory component in the U.S. National Health and Nutrition Examination Survey (NHANES): first-year results for measured olfactory dysfunction. Rev Endocr Metab Disord.

[23] Levis B, Benedetti A, Riehm KE, Saadat N, Levis AW, Azar M, Rice DB, Chiovitti MJ, Sanchez TA, Cuijpers P (2018). Probability of major depression diagnostic classification using semi-structured versus fully structured diagnostic interviews. Br J Psychiatry.

[24] Kroenke K, Spitzer RL, Williams JB (2001). The PHQ-9: validity of a brief depression severity measure. J Gen Intern Med.

[25] Ba DM, Gao X, Al-Shaar L, Muscat JE, Chinchilli VM, Beelman RB, Richie JP (2021). Mushroom intake and depression: a population-based study using data from the US National Health and Nutrition Examination Survey (NHANES), 2005–2016. J Affect Disord.

[26] Wu Q, Yan Y, La R, Zhang X, Lu L, Xie R, Xue Y, Lin C, Xu W, Xu J et al. Association of reproductive lifespan and age at menopause with depression: Data from NHANES 2005–2018. J Affect Disord 2024. 10.1016/j.jad.2024.04.077 38657760

[27] Liu Z, Fan Q, Wu S, Wan Y, Lei Y (2021). Compared with the monocyte to high-density lipoprotein ratio (MHR) and the neutrophil to lymphocyte ratio (NLR), the neutrophil to high-density lipoprotein ratio (NHR) is more valuable for assessing the inflammatory process in Parkinson’s disease. Lipids Health Dis.

[28] Huang AA, Huang SY (2023). Use of machine learning to identify risk factors for insomnia. PLoS ONE.

[29] Ale L, Gentleman R, Sonmez TF, Sarkar D, Endres C. nhanesA: achieving transparency and reproducibility in NHANES research. *Database (Oxford)* 2024, 2024. 10.1093/database/baae028 PMC11020206 38625809

[30] Meng F, Yan X, Qi J, He F (2022). Association of neutrophil to lymphocyte ratio, platelet to lymphocyte ratio, and monocyte to lymphocyte ratio with depression: a cross-sectional analysis of the NHANES data. J Affect Disord.

[31] Shin HY, Kang G, Kang HJ, Kim SW, Shin IS, Yoon JS, Kim JM (2016). Relationships between high-density lipoprotein cholesterol and depressive symptoms: findings of the Korean National Health and Nutrition Examination Survey (KNHANES). Psychiatry Res.

[32] Chen J, Huang Y, Li X (2024). The association between lymphocyte to high-density lipoprotein ratio and depression: data from NHANES 2015–2018. Brain Behav.

[33] Herman FJ, Simkovic S, Pasinetti GM (2019). Neuroimmune nexus of depression and dementia: Shared mechanisms and therapeutic targets. Br J Pharmacol.

[34] Bentivoglio M, Mariotti R, Bertini G (2011). Neuroinflammation and brain infections: historical context and current perspectives. Brain Res Rev.

[35] Irwin MR, Cole SW (2011). Reciprocal regulation of the neural and innate immune systems. Nat Rev Immunol.

[36] Schauer C, Janko C, Munoz LE, Zhao Y, Kienhöfer D, Frey B, Lell M, Manger B, Rech J, Naschberger E (2014). Aggregated neutrophil extracellular traps limit inflammation by degrading cytokines and chemokines. Nat Med.

[37] Kettenmann H, Hanisch UK, Noda M, Verkhratsky A (2011). Physiology of microglia. Physiol Rev.

[38] Miller AH, Raison CL (2016). The role of inflammation in depression: from evolutionary imperative to modern treatment target. Nat Rev Immunol.

[39] Gardner A, Boles RG (2011). Beyond the serotonin hypothesis: mitochondria, inflammation and neurodegeneration in major depression and affective spectrum disorders. Prog Neuropsychopharmacol Biol Psychiatry.

[40] Felger JC, Miller AH (2012). Cytokine effects on the basal ganglia and dopamine function: the subcortical source of inflammatory malaise. Front Neuroendocrinol.

[41] Bonacina F, Pirillo A, Catapano AL, Norata GD. HDL in Immune-inflammatory responses: implications beyond Cardiovascular diseases. Cells 2021, 10(5). 10.3390/cells10051061 PMC8146776 33947039

[42] Mazzuferi G, Bacchetti T, Islam MO, Ferretti G (2021). High density lipoproteins and oxidative stress in breast cancer. Lipids Health Dis.

[43] Gómez Rosso L, Lhomme M, Meroño T, Sorroche P, Catoggio L, Soriano E, Saucedo C, Malah V, Dauteuille C, Boero L (2014). Altered lipidome and antioxidative activity of small, dense HDL in normolipidemic rheumatoid arthritis: relevance of inflammation. Atherosclerosis.

[44] Kiecolt-Glaser JK, Derry HM, Fagundes CP (2015). Inflammation: depression fans the flames and feasts on the heat. Am J Psychiatry.

[45] Tran-Dinh A, Levoye A, Couret D, Galle-Treger L, Moreau M, Delbosc S, Hoteit C, Montravers P, Amarenco P, Huby T et al. High-density lipoprotein therapy in stroke: evaluation of endothelial SR-BI-Dependent Neuroprotective effects. Int J Mol Sci 2020, 22(1). 10.3390/ijms22010106 PMC7796353 33374266

[46] Yamada H, Umemoto T, Kawano M, Kawakami M, Kakei M, Momomura SI, Ishikawa SE, Hara K (2017). High-density lipoprotein and apolipoprotein A-I inhibit palmitate-induced translocation of toll-like receptor 4 into lipid rafts and inflammatory cytokines in 3T3-L1 adipocytes. Biochem Biophys Res Commun.

[47] Wang SH, Yuan SG, Peng DQ, Zhao SP (2012). HDL and ApoA-I inhibit antigen presentation-mediated T cell activation by disrupting lipid rafts in antigen presenting cells. Atherosclerosis.

[48] Sebastião AM, Colino-Oliveira M, Assaife-Lopes N, Dias RB, Ribeiro JA (2013). Lipid rafts, synaptic transmission and plasticity: impact in age-related neurodegenerative diseases. Neuropharmacology.

[49] Czysz AH, Rasenick MM (2013). G-protein signaling, lipid rafts and the possible sites of action for the antidepressant effects of n-3 polyunsaturated fatty acids. CNS Neurol Disord Drug Targets.

[50] Maes M, Plaimas K, Suratanee A, Noto C, Kanchanatawan B. First Episode Psychosis and Schizophrenia are systemic Neuro-Immune disorders triggered by a biotic stimulus in individuals with reduced Immune Regulation and Neuroprotection. Cells 2021, 10(11). 10.3390/cells10112929 PMC8616258 34831151

[51] Dahan S, Bragazzi NL, Yogev A, Bar-Gad M, Barak V, Amital H, Amital D (2018). The relationship between serum cytokine levels and degree of psychosis in patients with schizophrenia. Psychiatry Res.

[52] Howes OD, McCutcheon R (2017). Inflammation and the neural diathesis-stress hypothesis of schizophrenia: a reconceptualization. Transl Psychiatry.

[53] Horwitz AG, Zhao Z, Sen S (2023). Peak-end bias in retrospective recall of depressive symptoms on the PHQ-9. Psychol Assess.

[54] Trivedi MH, Rush AJ, Wisniewski SR, Nierenberg AA, Warden D, Ritz L, Norquist G, Howland RH, Lebowitz B, McGrath PJ (2006). Evaluation of outcomes with citalopram for depression using measurement-based care in STAR*D: implications for clinical practice. Am J Psychiatry.

[55] Huang AA, Huang SY (2023). Increasing transparency in machine learning through bootstrap simulation and Shapley additive explanations. PLoS ONE.

[56] Huang AA, Huang SY (2023). Hospitalized COVID-19 patients with diabetes have an increased risk for pneumonia, intensive care unit requirement, intubation, and death: a cross-sectional cohort study in Mexico in 2020. Health Sci Rep.

[57] Huang AA, Huang SY (2023). Quantification of the relationship of pyridoxine and spirometry measurements in the United States Population. Curr Dev Nutr.

